# Validity and reliability of a ruler drop test to measure dual-task reaction time, choice reaction time and discrimination reaction time

**DOI:** 10.1007/s40520-024-02726-6

**Published:** 2024-03-07

**Authors:** Soraia Ferreira, Armando Raimundo, Jesus del Pozo-Cruz, Nilton Leite, Ana Pinto, José Marmeleira

**Affiliations:** 1 Departamento de Desporto e Saúde, Escola de Saúde e Desenvolvimento Humano, Universidade de Évora, Largo dos Colegiais, Évora, 7000-727 Portugal; 2 Comprehensive Health Research Centre (CHRC), Palácio do Vimioso, Évora, 7002-554 Portugal; 3 Department of Physical Education and Sports, University of Seville, Sevilla, 41013 Spain; 4 Epidemiology of Physical Activity and Fitness across Lifespan Research Group (EPAFit), University of Seville, Sevilla, 41013 Spain

**Keywords:** Older adults, Ruler drop test, Simple reaction time, Choice reaction time, Dual-task reaction time, Discrimination reaction time

## Abstract

**Background:**

The aim of this study was to determine the absolute and relative reliability of the Ruler Drop Test (RDT) for assessing dual-task, choice, and discrimination reaction time. In addition, the construct validity of the RDT is examined in comparison to the Deary-Liewald reaction time (DLRT).

**Methods:**

Tests were administered by the same evaluator, one week apart. Intraclass Correlation Coefficient (ICC3.1) was used to measure relative reliability, and the standard error of measurement (SEM) and minimal detectable change (MDC95) were used to measure absolute reliability. Spearman correlation test was used to measure construct validity.

**Results:**

The results showed that the relative reliability was good for the choice ruler drop (ICC = 0.81), moderate for the dual-task ruler drop test (ICC = 0.70) and discrimination ruler drop test (ICC = 0.72), and good for simple ruler drop test. However, the simple ruler drop test had poor reliability (ICC = 0.57). The RDT shows construct validity compared to the DLRT.

**Conclusion:**

We conclude that the RDT is a suitable instrument for measuring dual-task, choice and discrimination reaction time. Future studies should explore the reliability of these measures in other populations.

## Introduction

Reaction time (RT) is the time interval between the presentation of a stimulus and the onset of the muscular response to that stimulus [1]. Reaction time is a widely used variable to measure the performance of human motor skills. The three most common types of RT are simple reaction time (SRT), choice reaction time (CRT), and discrimination reaction time (DRT) [2]. In SRT, there is a single stimulus and the participant must respond to that stimulus as quickly as possible [3]. In choice reaction time, there is more than one stimulus and for each stimulus there is a specific response. Finally, in the DRT, the participant has multiple stimuli but only one correct response. The participant must ignore the other stimuli and select the correct response [2, 3].

Over the years, research on RT has increased, especially in the field of gerontology, and it is known that RT is related to age, that it is associated with measures of higher cognitive function, and that it may be an indicator of an individual’s neurological status [4]. From the age of 30, RT slows down, and this decline is more pronounced after the age of 60 [5]. This slowing of RT is even greater when tasks are more complex and require more cognitive/motor processing [6]. Increased RT has been associated with decreased stride length [7], slower walking [8], decreased mobility [9] decreased lateral stability [10], impaired stair negotiation [11], and balance disturbances [12]. Information processing ability is also associated with decreased balance in dual tasks. One study showed that older people who fell tended to stop when speaking, and this is related to a person’s ability to do two or more tasks at once [13]. Falls are also associated with the individual capacity for inhibition (responding to salient stimuli and overriding dominant behavioral responses), with people with weak inhibition ability being less able to respond to changes in the environment [14]. Dual tasking and inhibition are two aspects of activities of daily living that are associated with RT and, consequently, increased risk of falls. Assessment of the different types of RT is extremely important for maintaining quality of life. Currently, there are some tests to assess simple RT, such as the Ruler Drop Test (RDT-S) [15], Deary-Liewald reaction time task (DLRT) [16], Numbers-based reaction time box [17], MOART Reaction Time and Movement Time Panel (Lafayette Instruments), Jensen box [18], Vienna test system (VTS) [19], the Cambridge Neuropsychological Test Automated Battery (CANTAB) [20].

Most of these tests have a cost associated with them, which makes their acquisition difficult. In this research, we adapted a simple and free test, the RDT-S. To our knowledge, no study investigated the psychometric properties of the RDT-S for measuring tasks other than simple RT. Previous studies have shown that the simple RDT-S can be a reliable instrument for collecting RT data in older adults living in the community [21] and in older adults with and without cognitive impairment living in institutions [22]. To our knowledge, no study investigates the psychometric properties of adaptations of the RDT-S. Therefore, in the present study we adapted the RDT-S to assess choice, dual-task, and discrimination RT and examined its psychometric properties. In addition, the construct validity of the RDT-S (single, choice, dual task) versus the DLRT (single, choice, dual task) is also examined.

## Method

### Participants

Fifty-one volunteers living in Évora (Portugal) participated. The inclusion criteria were living in community-dwelling, age of 60 years or more and without cognitive impairment. The exclusion criteria were having low vision, cognitive impairment, or Parkinson’s disease. The study was publicized in the community, and those interested contacted the principal investigator. All individuals interested in participating in the study completed the data collection forms.

The Mini-Mental State Examination (MMSE) was applied to all participants to observe their cognitive status, with cut-offs of ≤ 15 points for illiterate persons, ≤ 22 for persons ranging from 1 to 11 years of school education and ≤ 27 points or persons with *>* 11 years of school education [23]. Table 1 shows the general characteristics of the participants by group and in total. There were no significant differences between men and women in age, body mass index (BMI), years of education, and in physiological variables such as systolic and diastolic blood pressure and resting heart rate. Participants in this study had a mean age of 72.67 ± 5.3 years and a low level of education (35.3% attended only elementary school). According to the American College of Sports Medicine (ACSM, 2020) criteria regarding BMI, 88.2% of the participants of this study were overweight and 62.7% had a waist circumference greater than the recommended value (102 cm for men and 88 cm for women).

**Table 1:** Descriptive characteristics of the participants

	Men (*n* = 22)	Women (*n* = 29)	Total (*n* = 51)	P*
Age (years)	72.95 (5.9)	72.46 (4.9)	72.67 (5.3)	0.814^a^
Weigh (kg)	74.40 (10.2)	69.22 (8.4)	73.06 (10.1)	0.002*^a^
Height (cm)	1.66 (0.1)	1.54 (0.1)	1.59 (0.1)	0.000*
BMI (kg/m2)	28.22 (3.4)	29.42 (3.7)	28.89 (3.6)	0.312^a^
Education (years)	8.45 (5.4)	7.50 (4.1)	7.91 (4.7)	0.615
SBP (mm Hg)	136.6 (18.6)	133.15 (18.3)	134.65 (18.3)	0.553
DBP (mm Hg)	83.2 (15.6)	77.19 (8.2)	79.80 (12.2)	0.108
Waist circumference (cm)	101.50 (9.2)	96.32 (9.8)	98.6 (9.8)	0.055^a^
Hip circumference (cm)	99.29 (5.7)	105.24 (7.5)	102.7 (7.3)	0.005*

*Note* Data are expressed as mean ± standard deviation. BMI, body mass index; SBP, systolic blood pressure; DBP, diastolic blood pressure

^a^ANOVA Test p-value

^b^Paired-Samples T Test p-value

*p ≤ 0.05

All participants were informed of the aims of the study and gave informed consent before participation. The study was approved by the Ethics Committee of the University of Évora and conducted in accordance with the Declaration of Helsinki.

### Procedure

In this study, to assess the construct validity and inter-session test-retest reliability, the RT tests were administered twice, one week apart. At both evaluation occasions, the same kinesiologist (with extensive experience evaluating physical performance in older persons) collected the data. Both experiments were carried out independently in a silent room at the university. Participants briefly familiarized themselves with the tests and returned to the laboratory a week later to begin data collection.

### Instruments

#### Ruller drop test

##### Simple ruller drop test (RDT-S)

**Fig. 1:**
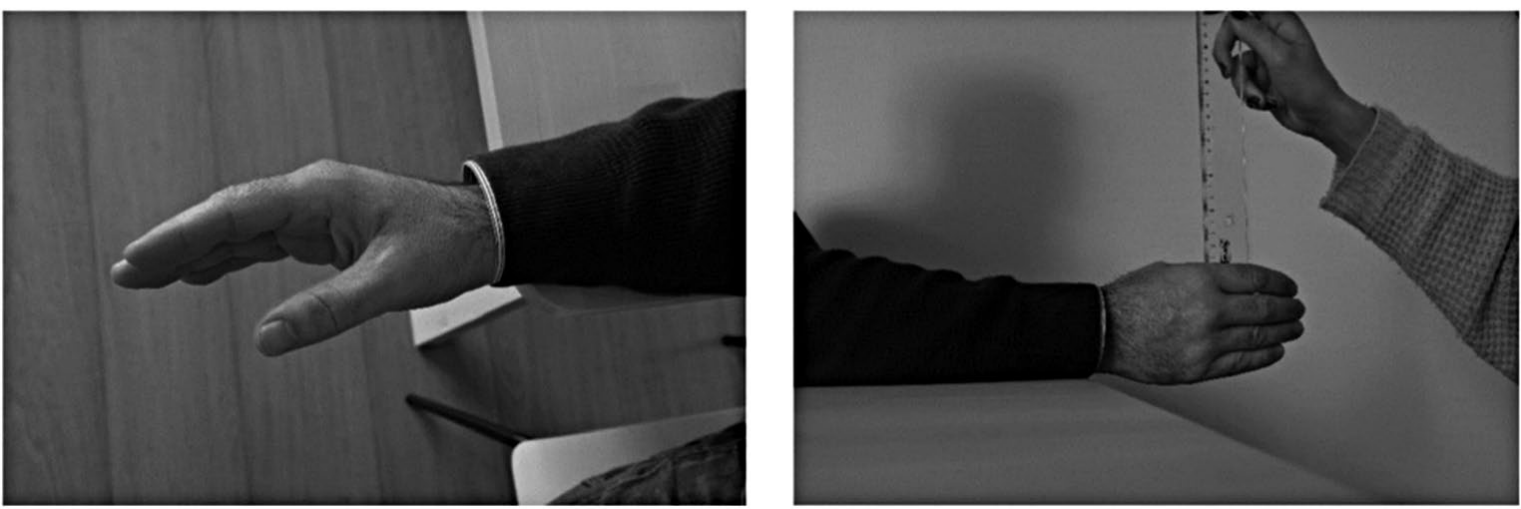
Ruler drop test, simple testing procedure

This test was based on previous studies in which the participant attempted to pick up the ruler as quickly as possible [15, 24]. The present study followed the protocol of a previous study [22]. The participant seated parallel to a table with the elbow bent at a 90° angle and leaning on it. The researcher held the 60 cm long ruler vertically and aligned the zero point with the top of the thumb and index finger (Fig. 1). The goal was for the participants to try to pick up the ruler as quickly as possible as soon as they saw it fall. The protocol was first explained by the evaluator. The participant was instructed to look at his/her hand and that the ruler could drop at any time after hearing the word “ready. Then the participants performed 3 practice trials to familiarize with the protocol. Afterwards, each participant performed the test 5 times, with the ruler falling at different times. To ensure that the time interval was the same for all participants in the test and retest, the evaluator used a metronome programmed to 60 bpm. A sequence was established in which the ruler was dropped after 3 s (first trial) 4s (second trial) 6s (third trial), 2s (fourth trial), and 4s (fifth trial) after the evaluator says “ready”. The sound of the metronome was heard only by the evaluator (using earbuds), On each trial, the value between the thumb and index finger was recorded. When the ruler fell to the floor, the value 61 cm (+ 1 cm than the length of the ruler) was recorded.

##### Ruler drop test with dual-task (RDT-DT)

This task was similar to the previous one, with the addition that the participant had to count backwards from two, starting at 100. The protocol was first demonstrated by the evaluator, and the evaluator started counting down at the number 150, every 2. After the demonstration, the participant performs some trials and starts counting down at 150. If the participant did not understand the exercise, he/she perform more trials. For assessment, the participant start counting at the number 100, and the evaluator drop the ruler with the following interval times: 3 s - first trial, 4s - second trial, 6s - third trial, 2s - fourth trial, and 4s - fifth trial. The value at which the participant grasped the ruler, the last number counted by the participant, and the number of errors (the number of mistakes he made while counting) were recorded. The exercise was audio-recorded so that the errors and the number the participant reached could be heard later. As described in the RDT-S protocol, a metronome and a 60 cm ruler were used to perform the test.

##### Choice ruler drop test (RDT-C)

**Fig. 2:**
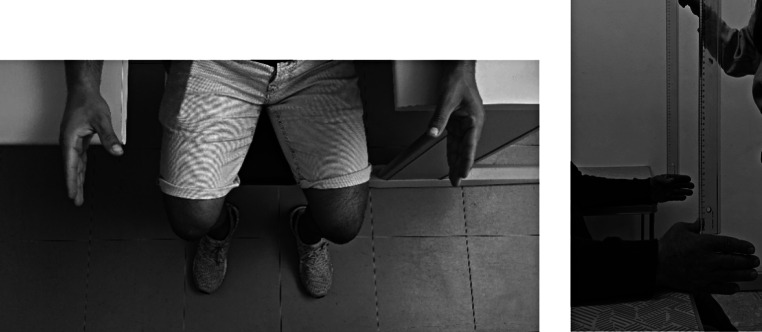
Choice Ruler drop test procedure

This test was designed to measure choice reaction time. The participant was seated parallel to two tables, one on right side and one on left side. The participant placed each arm on the respective table with the elbow bent 90 degrees and resting on the table. The researcher held two rulers perpendicular to the participant’s hands and aligned the zero point of each ruler with the top edge of the thumb and index finger (of the left and right hands) (Fig. 2).

The participant was asked to catch the dropped ruler as quickly as possible without closing the other hand. Two 60 cm long rulers were used. As in the previous protocols, a metronome was used to drop the rulers at the same time (Table 2). The participant did not know which ruler would fall (right or left), nor how long it would take after the word “ready” was spoken by the evaluator. Each time the participant closed the opposite hand where the ruler had fallen, it was considered an error. Initially, 3 trials were randomized for each hand. If the participant did not understand the exercise or was unsuccessful on the trials, additional trials were conducted until the participant understood and could perform the test. On each trial, we recorded how long the participant held the ruler. If the participant closed their hand against the fall of the ruler, this was considered an error and a value of 61 cm was assigned.

**Table 2:** Time intervals between trials used in the metronome for the RDT-C

Trials	1º	2º	3º	4º	5º	6º	7º	8º	9º	10º
Hand	NDH	DH	NDH	NDH	DH	NDH	DH	DH	NDH	DH
Time	3s	6s	4s	2s	4s	4s	3 s	4 s	6 s	2s

*Note* DH, Dominant Hand; NDH, Non- Dominant Hand

##### Discrimination ruler drop test (RDT-D)

This task aims to assess DRT, where there is multiple stimuli, and the participant must respond to only one. In this task, participants place their dominant arm on the table and follow the same protocol as in the RDT-S. Participants were instructed by the evaluator to grasp the ruler only when they hear the letter B, i.e., the evaluator could say three letters, A, B, or C, but if he says the letters A and C, the participants should not catch the ruler. The investigator must be trained beforehand so that the letter is pronounced at the same time as drops the ruler. The participant performs 5 practice trials in which the researcher says the different letters and drops the ruler. If the participant did not understand the task or was unsuccessful, it does a few more trials until the participant is sure the participant understood. The ruler used was the same as the other tests and whenever the ruler dropped at letter B, a value of 61 cm was assigned. If the participant held the ruler at letters A and C, this was counted as an error. The order and the time the ruler fell was the same for all participants (Table 3). Median were used for data analysis.

**Table 3:** Letter and time intervals between trials used in the metronome for the RDT-D

Trials	1º	2º	3º	4º	5º	6º	7º	8º	9º	10º
Letter	B	C	A	B	B	C	B	A	B	A
Time	3s	6s	4s	2s	4s	4s	3 s	4 s	6 s	2s

#### Deary-liewald reaction time task (DLRT)

Simple reaction time, SRT in dual-task conditions, and CRT were measured using the DLRT, which we named DLRT-S, DLRT-SDT and DLTR-C. The DLRT-S required participants to press the spacebar key in response to a single stimulus. A cross appears in a white square on the computer screen and the participant was instructed to click as fast as possible as soon as he sees the cross. After pressing the key, the cross disappears and reappears after a short period of time. It was specified that the time interval between stimuli could vary randomly between 1 and 3 s. The task included 2 trials and 20 tasks. The median was calculated for each participant. A colored paper was pasted so that participants could distinguish the space bar key from the other keys. For DLRT-SDT, SRT was measured as in the previous test, but a dual task was added, in which the participant was instructed to count from 2 to 2, starting in 0. Counting was started when the test began and ended when the test was completed. The number that the participant reached, and the number of errors was counted. Median and the number of errors were used for data analysis.

In the DLTR-C, there were four possible stimuli and only one correct answer for each of them. On the computer screen, against a blue background, there were four white squares centered and aligned horizontally. The cross could appear in any of the squares, with the “z” key corresponding to the leftmost square, the “x” key to the next square, the “comma” key to the third square, and the “dot” key to the rightmost square. Since participants had difficulty fixing the keys needed for the test, a black paper was placed over them (see figure). The cross disappeared when the participants selected one of the keys. If the participant selected the wrong key, the software counted an error. The interval between stimuli varied from 1 to 3 s, with the cross appearing randomly. Participants performed 4 practice trials and 20 test trials. Median were used for data analysis.

### Data analysis

Normality of all variables was tested with the Kolmogorov-Smirnov test. The relative reliability of the test-retest was determined by the two-way mixed model Intraclass Correlation Coefficient (ICC) [1, 3] with absolute agreement [25]. We used the following cut-off values; ICC > 0.90 excellent reliability; 0.75 to 0.90 good reliability; 0.50 to 0.74 moderate reliability; < 0.50 poor reliability [26, 27]. The standard error of measurement (SEM) [28] and minimum detectable change (MDC) [29], were calculated to determine absolute reliability. The calculation of SEM was determined by the formula, SEM = SD√(1-ICC) (SD is the average of the standard deviations of the two trials [30]). The criterion SEM < SD/2 was used to understand whether the accuracy of the measurement was acceptable [31]. The MDC was calculated for a confidence level of 95%, using the formula MDC_95_ = 1.96 × √2 x SEM [32]. The number of participants was calculated according to Walter et al. (1998) considering α = 0.05 and β = 0.20, where the desired ICC was 0.9, with a CI of 0.80. According to the calculation, the required sample size was 46. Construct validity between the RDT-S and DLRT was tested using the Spearman correlation coefficient, and data from the first session (after familiarization) were used. Data were analyzed using SPSS 24.0 for Windows (SPSS Inc., Chicago, Illinois), and the significance level was set at *p* < 0.05.

## Results

Fifty-one elderly people living in the community participated in this study, 22 men and 29 women. Three participants (two men and one woman) did not perform the RDT-C because they reported to be unable of seeing the two rulers simultaneously. Two participants in the RDT-DT, could not perform the 2-by-2 countdown, probably due to their low educational level. Therefore, the task was adapted for these participants, and a progressive 2-by-2 countdown was performed. The results of the relative and absolute reliability of the RT tests can be found in Table 4.

**Table 4:** Test-retest reliability of the reaction time tests in older adults

		Mean (SD)						
	n	Test	Retest	ICC (95%)	SEM	%	MDC_95_	%
RDT-S	51	31.99 (5.8)	30.35 (6.2)	0.573 (0.26–0.75)	3.9	12.5	10.75	34.5
RDT-C Error Total	48	1.42 (1.4)	1.06 (1.2)	0.743 (0.54–0.86)	0.66	53.1	1.82	146.5
RDT-C Error DH	48	0.77 (0.9)	0.65 (0.9)	0.693 (0.45–0.83)	0.52	73.7	1.44	203.3
RDT-C Error NDH	48	0.63 (0.8)	0.42 (0.7)	0.477 (0.08–0.704)	0.54	103.3	1.48	284.9
RDT-C	48	43.33 (9.82)	40.85 (9.66)	0.813 (0.66–0.89)	4.21	10	11.61	27.6
RDT-DT	51	36.69 (7.8)	35.74 (8.7)	0.701 (0.48–0.83)	4.51	12.4	12.43	34.3
RDT-DT Error	51	2.53 (2.5)	1.84 (2.1)	0.699 (0.47–0.83)	1.25	57.4	3.46	158.2
RDT-DT Nº	51	33.24 (22.9)	32.08 (21.5)	0.801 (0.65–0.89)	9.91	30.4	27.34	83.7
RDT-D	51	32.69 (11.0)	28.68 (8.8)	0.718 (0.47–0.85)	5.25	17.1	14.49	47.2
RDT-D error	51	1.57 (1.3)	1.14 (0.8)	0.450 (0.07–0.68	0.78	57.8	2.16	159.5

*Note* RDT-S, simple ruler drop test; RDT-C, choice ruler drop test; RDT-DT, dual-task ruler drop test; RDT-D, discrimination ruler drop test; DH, dominant hand; NDH, non- dominant hand; SD, standard deviation; ICC, intraclass correlation coefficient; SEM, standard error of measurement; MDC, minimal detectable change

Relative reliability was good for the RDT-C and the number achieved in RDT-DT (ICCs = 0.81 and 0.80, respectively) and moderate for the RDT-S, total RDT-C errors, RDT-C dominant hand errors, RDT-DT, the number of errors in RDT-DT, and the RDT-D (ICCs = 0.57, 0.74, 0.69, 0.70, 0.69 and 0.71, respectively). The number of errors in the RDT-C for the non-dominant hand and the errors in the RDT-D showed poor reliability (ICCs = 0.48 and 0.45, respectively). The SEM values were 12.5% on the RDT-S, 10% on the RDT-C, 12.4% on the RDT-DT and 17.1% on RDT-D. The MDC_95_ values were 34.5%, 27.6%, 34.3% and 47.2% on RDT-S, RDT-C, RDT-DT and RDT-D, respectively.

Spearman correlation between RDT-S and DLRT performance is shown in Table 5. Significant associations were found between RDT-S and DLRT-S (*r* = 0.423; *p* = 0.002), RDT-C and DLRT-C (*r* = 0.375; *p* = 0.006), and RDT-DT and DLRT-DT (*r* = 0.025; *p* = 0.025).

**Table 5:** Spearman correlation between reaction time variables

	DLRT-S	DLRT-SDT	DLRT-C	
RDT-S	0.423**	0.309*	0.267	
RDT-DT	0.375**	0.322**	0.375**	
RDT-C	0.167	0.215	0.394**	
RDT-D	0.375**	0.375**	0.381**	

*Note* RDT-S, simple ruler drop test; RDT-C, choice ruler drop test; RDT-DT, dual-task ruler drop test; RDT-D, discrimination ruler drop test; DLRT-S, simple Deary-Liewald reaction time task; DLRT-SDT, dual-task Deary-Liewald reaction time; DLRT-C, choice Deary-Liewald reaction time task

*correlation is significant at the 0.05 level

**correlation is significant at the 0.01 level

## Discussion

The original RDT-S has been used as a measure of SRT [33], and it is a simple and quick test to perform. In this study, we adapted the RDT-S to measure SRT with dual task, CRT and DRT and examined the relative and absolute reliability of these three variants. This study also focused on the construct validity of the RDT-S, considering DLRT as a reference.

The ICC for the RDT-S was moderate (ICC = 0.57). In contrast, the study by van Schooten et al. [21] showed an ICC of 0.98. This discrepancy in results could be related to the methodology used in each study. The study of van Schooten used an electronic device that allowed measurement of acceleration between the moment that the device was dropped and the moment it was picked up by the participant. Another study developed with elderly people attending institutions showed good reliability of the RDT-S for elderly without cognitive impairment (ICC = 0.84) and with cognitive impairment (ICC = 0.80) [22].

SEM and MDC_95_ for the RDT-S, were relatively low (SEM = 3.90 and MDC_95_ = 10.75). The SEM was lower than the SEM < SD/2 criterion (5.59/2 = 2.80), suggesting acceptable measurement accuracy for this test.

The DLRT-C performance was evaluated by the number of errors and the median of values corresponding to the centimeters at which the ruler was catch by the participant. The RDT-C showed good reliability (ICC = 0.81), but the number of errors showed moderate (error of both hands, ICC = 0.74; error with the right hand, ICC = 0.69) and low (error of the non-dominant hand, ICC = 0.48) reliability. The absolute reliability for the RDT-C was slightly higher than for the RDT-S (SEM = 4.21 e MDC_95_ = 11.61). The SEM value was above the criterion, SEM < SD/2 (9.74/2 = 4.87), which indicates that the measurement accuracy can be considered unacceptable. Furthermore, according to the MDC_95_ values, participants must improve their performance by 11.6 cm to achieve a real change in performance. These results imply that participants must improve by more than 27% compared with their current results. The RDT-C had lower reliability than the RDT-S, which may be related to the loss of peripheral vision associated with age. In our study, 3 participants were unable to complete the task because of their low peripheral vision. On the other hand, the development of a test that assesses CRT with only two stimulus responses might allow the inclusion of a larger number of participants with different pathologies.

In the RDT-DT, relative reliability was good with an ICC = 0.70. The SEM suggests an acceptable measurement accuracy that meets the above criterion (8.25/2 = 4.12). On the other hand, the MDC_95_ shows high scores that require an improvement of 12.43 cm to achieve a real change in performance. Participants must be able to improve their performance by 34.3% for it to be a real change. At RDT-DT, we also examined the number of counting errors participants made and the number they achieved at the end of the exercise. The reliability on the number of errors the participant makes during the test is moderate (ICC = 0.70), and to achieve a real change in the test, they need to make on average 3.5 fewer errors during the test.

On the RDT-D, relative reliability was moderate for task performance and poor for the number of errors made by the participant (RDT-D, ICC = 0.72; RDT-D errors, ICC = 0.45). In this test, if the participant picks up the ruler when the rater says the letter A or C, it is considered an error. A previous study [21] examined DRT using a test similar to the ruler drop test. The van Schooten at al. study used a device that contained an accelerometer and displayed a green light at specific times. The participant was instructed to hold the device when the light illuminated and to suppress the response when the light did not illuminate. The participant was instructed to pick up the device when a green LED lit. If the LED did not light up, the participant was to drop the LED on the floor. The results of this study are consistent with ours, showing moderate relative reliability (ICC = 0.72). Our study also assessed the absolute reliability of the RDT-D, and the number of errors is relatively low (RDT-D, SEM = 5.25; RDT-D error, SEM = 0.78), with SEM suggesting acceptable measurement accuracy. MDC_95_ suggests that participants need to grasp the ruler 14.5 cm earlier to make a real change; this is the highest value in our tests.

This study also assessed the construct validity of RDT-S and DLRT. The second assessment time point was used to control for the learning effect. Our results show significant correlations when we compare the RDT-S with the DLRT. We associated the RDT-S and the DLRT-S, the RDT-DT and the DLRT-DT, and the RDT-C and the DLRT-C. Our results are aligned with the original DLRT article, where a significant correlation was ere reported between the DLRT and the number-box test [16]. Although the tests used in the original article and in the present study are different, the correlations remain significant. It is important to keep in mind that all versions of the RDT-S used in this study were analog, i.e., performed manually by the researcher. To reduce the influence of the rater’s behavior, a metronome was used in all tests, which allowed for greater homogeneity in the performance of the tests.

Overall, test performance on the RDT-S, RDT-C, and RDT-D was significantly better on the second trial than on the first. These results could be related to the fact that the participants felt more confident in experimenting. However, a moment of familiarization was performed, so the learning effect was minimized. On the RDT-DT, there was no significant difference between the first and the second experiment. This could be related to the fact that a cognitive task was given, which increased the complexity of the task. This study shows that the RDT-C, the RDT-DT, and the RDT-DT are reliable in community-dwelling older people. With these tests, we can evaluate the different types of RT in a simplified way and with little material. Another advantage is that evaluations can be performed in different contexts without having to be in a laboratory or use expensive equipment.

This study has some limitations. First, individuals with low peripheral vision were unable to perform the test that assesses CRT. Another limitation is that our tests were performed on elderly people living in the community-dwelling and leading an active lifestyle, and our results may not be generalizable to people living in nursing homes. Finally, if a ruler made of different materials (e.g. aluminum) had been used, the reaction time results might have differed. In the future, it will be important to investigate whether there are differences in the results of the ruler drop test when using rulers made of different materials.

## Conclusion

This study showed that RDT-C, RDT-DT, and RDT-D had moderate to good reliability. Measurement accuracy was acceptable for RDT-S, RDT-DT, and RDT-D, although the same did not happen for RDT-C. All tests have been successfully executed for the study population, with RDT-C having some limitations for individuals with lower peripheral vision. Future studies should investigate alternatives to measure choice RT. The use of this instrument may be useful in assessing processing speed in older people, informing intervention for acting on their maintenance/improvement in a more practical and effective way.

## References

[1] Lord S, Delbaere K, Sturnieks D (2018) Aging. In Brian D. & Lord S (ed) Balance, Gait, and Falls. Elsevier, pp. 157–171.

[2] Magill R, Anderson DI (2017). Motor learning and control: concepts and applications.

[3] Stebbins G. Neuropsychological Testing. In: Goetz C, (ed) Textbook of Clinical Neurology: Third Edition. Elsevier, 2007. p. 1–1364.

[4] Salthouse T (2007) Reaction time. In: Birren J, (ed). Encyclopedia of Gerontology. Elsevier, pp 407–410

[5] Newell K, Vaillancourt D, Sosnoff J (2006) Aging, Complexity, and Motor Performance. In: Birren J, Schaie. Handbook of The Psychology of Aging. New York, pp 163-178

[6] Darbutas T, Juodžbalienė V, Skurvydas A, Kriščiūnas A (2013) Dependence of reaction time and movement speed on task complexity and age. Medicina 42(1): 18-22 23652713

[7] Callisaya ML, Blizzard L, Schmidt MD, Mcginley JL, Lord SR, Srikanth VK (2009). A population-based study of sensorimotor factors affecting gait in older people. Age Ageing.

[8] Menz HB, Lord SR, Fitzpatrick RC (2003). Age-related differences in walking stability. Age Ageing março de.

[9] Lord SR, Murray SM, Chapman K, Munro B, Tiedemann A (2002) Sit-to-stand performance depends on sensation, speed, balance, and psychological status in addition to strength in older people. The journals of gerontology Series A, Biological sciences and medical sciences. 57: 539-543. 10.1093/gerona/57.8.m539 12145369

[10] Lord SR, Rogers MW, Howland A, Fitzpatrick R (1999). Lateral stability, sensorimotor function and falls in older people. J Am Geriatr Soc.

[11] Tiedemann AC, Sherrington C, Lord SR (2007) Physical and psychological factors associated with stair negotiation performance in older people. The journals of gerontology Series A, Biological sciences and medical sciences 62(11):1259–1265 10.1093/gerona/62.11.1259 18000146

[12] Sturnieks DL, Menant J, Vanrenterghem J, Delbaere K, Fitzpatrick RC, Lord SR (2012). Sensorimotor and neuropsychological correlates of force perturbations that induce stepping in older adults. Gait Posture julho de.

[13] Lundin-Olsson L, Nyberg L, Gustafson Y (1997) "Stops walking when talking" as a predictor of falls in elderly people. Lancet 349(9052):617 10.1016/S0140-6736(97)24009-2 9057736

[14] Nagamatsu LS, Munkacsy M, Liu-Ambrose T, Handy TC (2013). Altered visual-spatial attention to task-irrelevant information is associated with falls risk in older adults. Neuropsychologia dezembro de.

[15] Johnson BL, Nelson JK (1969). Practical measurements for evaluation in physical education. Fourth edi.

[16] Deary IJ, Liewald D, Nissan J (2011). A free, easy-to-use, computer-based simple and four-choice reaction time programme: the Deary-Liewald reaction time task. Behav Res Methods 24 De março de.

[17] Cox B, Huppert F, Whichelow M (1993). The health and lifestyle survey: seven years on.

[18] Jensen A (1987) Individual differences in the Hick paradigm. In: Vernon PA (ed) Speed of information-processing and intelligence. Ablex Publishing, pp. 101–175

[19] Schuhfried (2013). Vienna Test System: Psychological Assessment.

[20] Sahakian BJ, Owen AM (1992) Cambridge Neuropsychological Test Automated Battery (CANTAB): A factor analytic study of a large sample of normal elderly volunteers. Dementia 5;266-281 10.1159/000106735 7951684

[21] van Schooten KS, Duran L, Visschedijk M, Pijnappels M, Lord SR, Richardson J (2019). Catch the ruler: concurrent validity and test-retest reliability of the ReacStick measures of reaction time and inhibitory executive function in older people. 1 de agosto de.

[22] Ferreira S, Raimundo A, del Pozo-Cruz J, Marmeleira J (2021) Psychometric properties of a computerized and hand-reaction time tests in older adults using long-term facilities with and without mild cognitive impairment. Experimental gerontology 147. 10.1016/j.exger.2021.111271 33549821

[23] Guerreiro M, Silva A, Botelho M, Leitão O, Castro-Caldas A, Garcia C (1994). Adaptação à população Portuguesa Da tradução do Mini Mental State Examination (MMSE) – ScienceOpen. Rev Port Neurol.

[24] Del Rossi G, Malaguti A, Del Rossi S (2014). Practice effects associated with repeated assessment of a clinical test of reaction time. J Athl Train.

[25] Weir JP (2005). Quantifying test-retest reliability using the intraclass correlation coefficient and the SEM. J Strength Conditioning Res fevereiro de.

[26] Koo TK, Li MY (2016). A Guideline of selecting and reporting Intraclass correlation coefficients for Reliability Research. J Chiropr Med 1 de junho de.

[27] Portney LG, Watkins MP (2015) Foundations of clinical research: applications to practice. FA Davis

[28] Bruton A, Conway JH, Holgate ST (2000) Reliability: What is it, and how is it measured?. Physiotherapy 86(2):94–9

[29] Šerbetar I (2015) Establishing Some Measures of Absolute and Relative Reliability of a Motor Tests. Croatian Journal of Education 17: 37-48

[30] Hopkins WG (2000) Measures of reliability in sports medicine and science. Sports Med 30(1):1–15 10.2165/00007256-200030010-00001 10907753

[31] Ries JD, Echternach JL, Nof L, Blodgett MG (2009). Test-retest reliability and minimal detectable change scores for the timed Up &amp; Go Test, the six-Minute Walk Test, and Gait Speed in People with Alzheimer Disease. Phys Therapy 1 de junho de.

[32] Terwee CB, Bot SDM, de Boer MR, van der Windt DAWM, Knol DL, Dekker J (2007). Quality criteria were proposed for measurement properties of health status questionnaires. J Clin Epidemiol janeiro de.

[33] Johnson BL, Nelson JK (1986). Practical measurements for evaluation in Physical Education. Fourth edi.

